# Machine Learning-Enriched Lamb Wave Approaches for Automated Damage Detection

**DOI:** 10.3390/s20061790

**Published:** 2020-03-24

**Authors:** Zi Zhang, Hong Pan, Xingyu Wang, Zhibin Lin

**Affiliations:** Department of Civil and Environmental Engineering, North Dakota State University, Fargo, ND 58018, USA; Zi.zhang@ndsu.edu (Z.Z.); Hong.pan@ndsu.edu (H.P.); Xingyu.wang@ndsu.edu (X.W.)

**Keywords:** Lamb wave, data-driven approach, damage identification, structural health monitoring, machine learning

## Abstract

Lamb wave approaches have been accepted as efficiently non-destructive evaluations in structural health monitoring for identifying damage in different states. Despite significant efforts in signal process of Lamb waves, physics-based prediction is still a big challenge due to complexity nature of the Lamb wave when it propagates, scatters and disperses. Machine learning in recent years has created transformative opportunities for accelerating knowledge discovery and accurately disseminating information where conventional Lamb wave approaches cannot work. Therefore, the learning framework was proposed with a workflow from dataset generation, to sensitive feature extraction, to prediction model for lamb-wave-based damage detection. A total of 17 damage states in terms of different damage type, sizes and orientations were designed to train the feature extraction and sensitive feature selection. A machine learning method, support vector machine (SVM), was employed for the learning model. A grid searching (GS) technique was adopted to optimize the parameters of the SVM model. The results show that the machine learning-enriched Lamb wave-based damage detection method is an efficient and accuracy wave to identify the damage severity and orientation. Results demonstrated that different features generated from different domains had certain levels of sensitivity to damage, while the feature selection method revealed that time-frequency features and wavelet coefficients exhibited the highest damage-sensitivity. These features were also much more robust to noise. With increase of noise, the accuracy of the classification dramatically dropped.

## 1. Introduction

Civil engineering structures are the key backbone of the society and economy. Understanding structural performance, and assessing structural condition, and providing real-time decision making are crucial components in structural health monitoring (SHM), in order to avoid catastrophic events, and improve public safety [1]. As compared to conventionally vision-based techniques [2,3] or vibration-based techniques [4,5,6] that are mostly sensitive only to severe damage, guided wave-based techniques are often capable of identifying more minute damage and a tiny anomaly in active manner [7]. Guided waves display in different forms, such as the axial wave, flexural wave, shear wave, Rayleigh wave and Lamb wave. Among them Lamb waves exhibit their merits over other types for damage detection and classification, due to their strong penetration that can allow them to propagate in thin plate structure with a high attenuation ratio. Particularly, their two modes, symmetric mode and antisymmetric mode, could transfer to other types [8]. For instance, some researchers [7] found that at low frequency, the symmetric Lamb wave was similar with axial waves, while the antisymmetric Lamb wave was identical to flexural waves. When at high frequency, the speed of the Lamb wave can be similar to Rayleigh waves.

Much research has been conducted on the physics-based signal process of the Lamb wave for capturing delamination [9], monitoring fatigue damage and thermal damage in composite structures [10], identifying and locating the damage of metallic structures [11] and improving the effectiveness of the time-reversal damage detection procedure by single-mode [12]. Due to the complex underlying mechanism of the Lamb waves propagation and scattering, some data generated could be complicated to handle. For instance, Lamb wave propagations along a structural component could have virtually infinite combinations of structural shapes and damages, which could make it highly nontrivial to establish a fully physics-based predictive model [13,14]. In such a way, one attempt was to tackle them in a case-by-case manner, while with a loss of generality. Moreover, with inherent structural uncertainty, interpreting hidden information from signals could be more challenging. 

Clearly, such a complex signal process requires better data mining and information fusion. Alternatively, machine learning [15,16,17,18] and artificial intelligence methods [19] are rapidly evolving and leading to more accurate predictions for broader applications, great potential for accelerating a lamb-wave signal process and providing higher accuracy for damage detection. A literature review demonstrated that a few studies attempted to deploy the capability of machine learning for enhancing lamb wave-based damage detection. For instance, matching pursuit algorithms were introduced into this area by Wang [20], who designed a comprehensive dictionary to identify the location of damage. Yang and He [21] employed the Bayesian method to achieve crack size predictions accurately. Legendre et al. [22] presented an automated system to classify the metallic welds based on wavelet transform of Lamb wave by a neural network. Su et al. [23] used an artificial neural network (ANN) to establish a Lamb wave propagation based quantitative identification shame for delamination in composite structures. Das et al. [24] used a one-class support vector machine (SVM) technique to demonstrate damage classification. This method also demonstrated better classification in the presence of material and experimental uncertainties. Sun et al. [25] employed a Lamb wave damage quantification method using the least square support vector machine (LS-SVM) and genetic algorithm (GA). Three sensitive features were extracted from the Lamb wave signal: the normalized amplitude, phase change and correlation coefficient. GA was used for obtaining the optimal model parameters. Hossein et al. [26] applied multiclass SVM, classified the damage and estimated the severity of damages. Clearly, these studies mainly focused on certain features for damage detection, but none tried to discuss the impacts of the sensitivity of features used on the effectiveness of classification. In addition, structural uncertainty, such as noise interference, was considered less in their investigations. Many opportunities remain in SHM to accelerate the rate of knowledge discovery for more precise and accurate prediction.

Therefore, this study aimed to develop a holistic learning framework, a workflow from the dataset to damage-sensitive feature extraction and selection, for Lamb-wave-based damage detection. The datasets were generated by the Lamb wave signal through finite element simulation, in which different damage severities and orientations were specifically designed for generality. Wavelet coefficients and other forms were extracted and evaluated in terms of their damage sensitivity using feature selection criteria. Different noise levels were included to the received signals to simulate structural uncertainty. The shallow-learning algorithm, SVM, was then used for the learning model training for prediction, while a grid-search technique was used to optimize the SVM. Confusion metrics as well as feature scatting were presented to demonstrate the impacts of the features on the classification and identification of mechanical damage.

## 2. Machine Learning Enriched Lamb Wave Approaches

**Figure 1:**
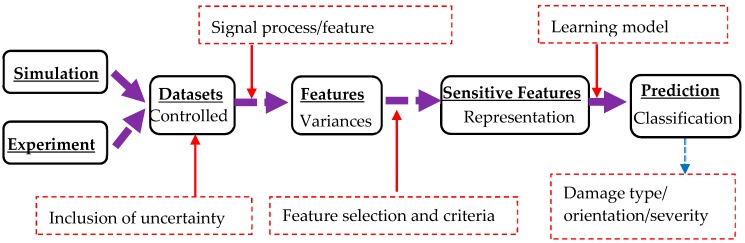
Framework of the machine learning-enriched method for damage detection.

This study aimed to propose machine-learning-enriched methods for damage detection. As illustrated in Figure 1, the learning framework consisted of (a) datasets collected from either experiment or simulation for mechanical damage/defects, (b) features generated by feature extraction for associated physics-based signal process, (c) sensitive features through feature selection and related criteria and (d) a prediction formulated by learning model training (e.g., shallow learning or deep learning) for knowledge discovery associated with mechanical damage if actual data are applied.

This learning framework provided a workflow from data to sensitive feature extraction. Although large datasets from different sources could enrich the extraction of representation, simulation using Lamb wave excitation was selected herein as a demonstration to generate data. Accordingly, a different signal process under frequency or time domains could be selected to filter data and extract features that could represent the hidden information associated with mechanical damage. To apply damage-sensitive features for data training, feature selection and criteria were determined in this study, while SVM learning algorithms were designed for learning model training, where the radial basis function (RBF) kernel was herein chosen as the kernel function, as presented in detail in the following sections. Note that deep learning algorithms, such as convolutional neural networks (CNN) or deep belief network, are powerful for automated feature extraction. Such functionality in the deep learning requires less or even no physics meaning during the feature extraction and feature selection, while one of the objectives in this study was to gain understanding of selective features provided in the shallow learning. Thus, the deep learning was not selected herein for such consideration.

To achieve that, Section 3 was to present Lamb-wave based damage detection in detail, including a brief summary of the signal process by basic lamb wave theory and simulation, while test scenarios were designed to account for different damage type, damage level and damage orientation. Section 4 discussed feature extraction methods using signal characteristics under time and frequency domains, while sensitive features to defects were extracted using feature selection methods for better data classification. The merits of using the proposed machine-learning-enriched methods over conventional physics-based signal process were mainly on:

(a) Handling nonlinear and high-dimensional features; physics-based features such as amplitude, phase change and correlation coefficient, which are often used explicitly for determining damage level and size, could be insensitive to defects in some cases when facing with the complexity of a Lamb wave multimodal interaction, noise or other interference. Differently, feature extraction and feature selection in the machine learning could effectively extract sensitive features for damage detection, with less physical representation, as discussed in Section 4.

(b) Tackling more structural complexity with less physical restraints; as stated in Section 3, Lamb wave exhibits non-stationary and nonlinear behavior, experiencing complex dispersion and coherent multimode interaction. Different to physics-based methods that attempt decomposition of mixed modes for signal process, such as using the first symmetric mode (*S*_0_) or antisymmetric mode (*A*_0_), the machine learning could extract sensitive damage features, with less or without such physical restraints. As a result, with representative data, the machine learning could provide better damage detection with minimized explicit formation that physics-based methods highly rely on. 

(c) Uncovering structural uncertainty; consider that engineering structures are often exposed to high levels of uncertainty, structural uncertainty is one of challenges for physics-based methods in SHM. The cases were designed in Section 6 to address this challenge and demonstrate the effectiveness of the proposed learning framework under structural uncertainty due to mixed data types, noise level and material discontinuity from weldment. The findings were expected to provide new vision using machine learning methods for engineering applications.

## 3. Datasets Generated from Lamb Wave Approaches

### 3.1. Concept of Lamb Wave Excitation

Lamb-wave based damage detection is a non-destructive strategy by identifying change of the wave form when the excited Lamb-wave signals encounter damage or other material discontinuity and quantifying their location and severity of the damage. Lamb wave excited in thin plate exhibits in different modes, symmetric mode (labeled as *S* mode) and antisymmetric mode (labeled as *A* mode), while each one has its own application for different types of damage [27]. For instance, the *S*_0_ mode is sensitively for internal damages in thin plate-like structure, while *A*_0_ mode is sensitively for surface damages [8]. Particularly, these two modes could carry more energy than others. The Lamb wave in a thin plate can be expressed as [28]:

(1) $$\frac{\tan\left(q\times h\right)}{\tan\left(p\times h\right)}=\frac{-4k^{2}pq}{{\left(q^{2}-k^{2}\right)}^{2}}\;(S\;\mathrm{mode})$$

(2) $$\frac{\tan\left(q\times h\right)}{\tan\left(p\times h\right)}=\frac{{\left(q^{2}-k^{2}\right)}^{2}}{-4k^{2}pq}\;(A\;\mathrm{mode})$$

where *k* is wavenumber, *f* is frequency and *h* represent the thickness. The Lamb wave speed is given by $c_{p}=\frac{\omega }{k}$, which is the velocity of individual waves.

**Figure 2:**
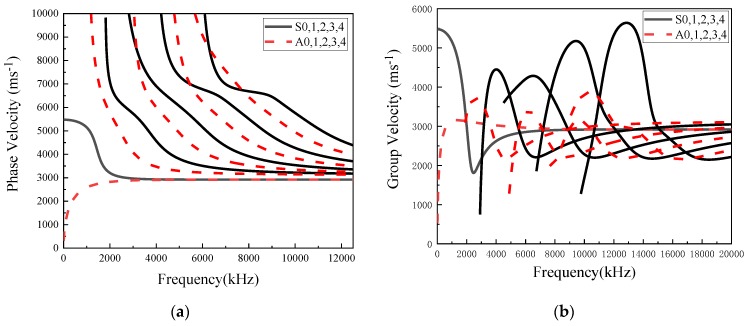
Dispersion curves of (**a**) phase velocity and (**b**) group velocity.

As such, Lamb wave phase velocities $C_{p}$ and group velocities *C_g_* of an aluminum plate are shown in Figure 2a,b, in which the modes increase with the increase of the higher frequency range. 

Pulse-echo method is one way to excite a Lamb wave, where two actuators are usually mounted at the same side of the plate for signal transmitting and receiving in process [29]. One is for the transmitter and the other services as receiver, as pulse-echo configuration. In this configuration, the signal is reflected when it reaches the boundary or damage of the plate, then, this echoed signal is captured by the receiver.

### 3.2. Simulation of Lamb Wave Excitation Along Structural Components

Lamb wave propagates in the plate as a pattern illustrated in Figure 2a,b, while different damage location and severity could cause the wave scattering in the form of mode conversion, as well as reflection and transmission. As a result, linking the changes of the wave modes with associated damage types, localization and severity make it flexible for damage identification. Much research [1,8] has demonstrated that the Lamb wave is highly sensitive to relatively tiny damage, which is often failed by other non-destructive detection methods. Additionally, the Lamb wave is robust since it can propagate to a large distance with little attenuation. In the context of the phase velocity of the Lamb wave, more wave modes are involved when the frequency of the wave is higher, which could lead to more complicatedly interacted modes. To make the signal more sensitive to damage, the frequency of the Lamb wave used in the excitation should be limited at the lower level (less than 1000 kHz) [1,8], which in turn leads to remaining the lowest modes, *S*_0_ and *A*_0_. 

**Figure 3:**
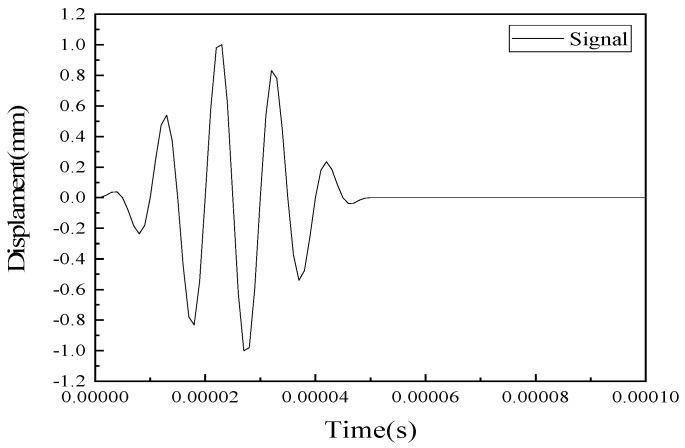
Excited Lamb wave in the simulation.

As such, the commercially available finite element software, COMSOL^®^, was used in this study. As shown in Figure 3, the excitation signal with 100 kHz, *D*(*t*), was defined by a 5-cycle sine function operated with a Hanning window by the form:

(3) $$D\left(t\right)=A\left(1-cos\frac{2\pi f_{c}t}{n}\right)\sin\left(2\pi f_{c}t\right)$$

where *A* is amplitude of the signal, *f_c_* is the frequency and *n* is the number of the period. The signal was defined in COMSOL to simulate the effect of the actuator, while the displacement was set with opposite orientation at the ends of the piezo actuator.

### 3.3. Calibration of Simulation

**Figure 4:**
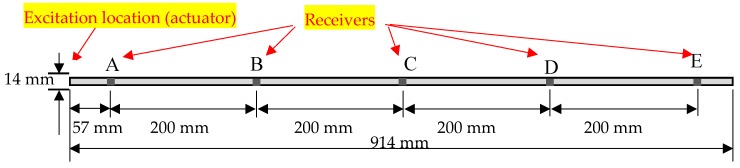
Aluminum beam.

This section was to calibrate the parameter used for modeling and characterizing Lamb wave propagation along a structure. A prototype of a thin narrow-strip aluminum beam was selected from the literature work by V. Giurgiutiu [30]. The plate had a dimension of 914 mm by 14 mm with a thickness of 1.6 mm. The piezo actuator was installed in front of the beam, as shown Figure 4. Five different spots, *A* to *E*, were selected to receive the signal, as shown in Figure 4. The damage was located at the point C (457 mm away from left side) of the plate. An 8-mm through-the-thickness notch was defined in the COMSOL to simulate the shape of the damage.

Free triangular elements available in COMSOL were used to mesh the aluminum plate. The maximum size of each element was 4.937 mm, and the minimum size was 2.468 mm. Boundary conditions were given as free. 

**Figure 5:**
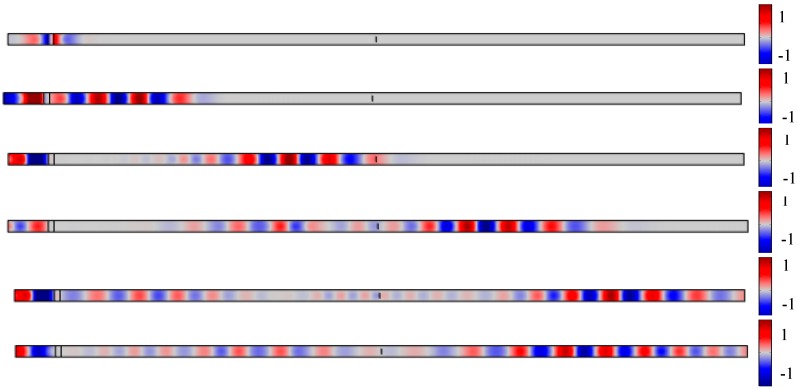
Wave propagation through the whole span.

**Figure 6:**
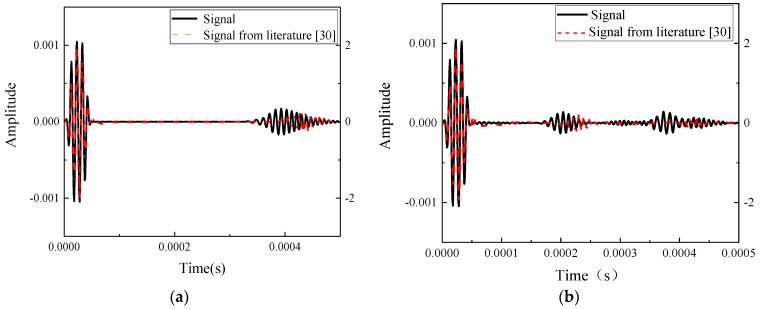
Comparison of the results. (**a**) Undamaged state predicted by the proposed work and the literature [30]; (**b**) Damage state with 8-mm crack predicted by the proposed work and the literature [30].

**Figure 7:**
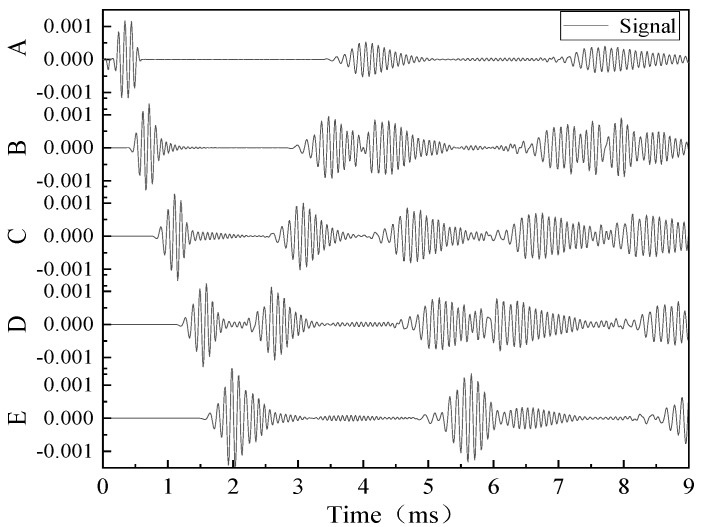
The signal received at sensors A-E predicted by the proposed work.

The results were plotted in Figure 5 to display Lamb wave propagating through the plate under the damage state. It gives a detail about the wave propagating in a different period. The excitation was sent into the plate at the left side and then the wave propagated to both sides. The wave returned when it arrived at the left boundary. At the other side, when the wave interacted with the damage, part of the wave reflected, and the rest continued to propagate forward. The echoed signals were accepted at location A. Figure 5 presents the results from the simulation comparing with the result in the literature [30]. Figure 6a is the signal under undamaged state, which has an initial signal at the front of the signal and an echoed signal located around 4e-4s. The amplitudes of the signals are different due to the different amplitude of the excitation. Figure 6b represents the results of an 8 mm crack. These two signals had a similar reflection at 2e-4s, suggesting that the signal received from the damage. Figure 7 displayed the signals received at sensors A to E. Note that there was a different in amplitude of the signals, the reason could be that the parameters of the signal and the material were not shown. The comparison verified the simulation used in this study was proper for Lamb wave simulation and thus the further exploration of simulation was used for the following parametric study and data generation.

### 3.4. Design of Scenarios and Data Augmentation

#### 3.4.1. Design of Scenarios 

Five different damage types were designed, including notch-shaped damage, circular-shaped damage, square-shaped damage, diamond-shaped damage and oval-shaped damage. All these through-the-thickness damages were located at the middle of the plate. The length of them was 6 mm equally. In addition, the notch-shaped damage was designed in 6 different sizes and 7 different orientations, similar to a crack. Therefore, the overall of 17 different states were designed and listed in Table 1. The excitation was a 5-cycle tone burst with a Hanning window of frequency of 100 kHz used for the model.

#### 3.4.2. Data Augmentation and Noise Interferences 

**Figure 8:**
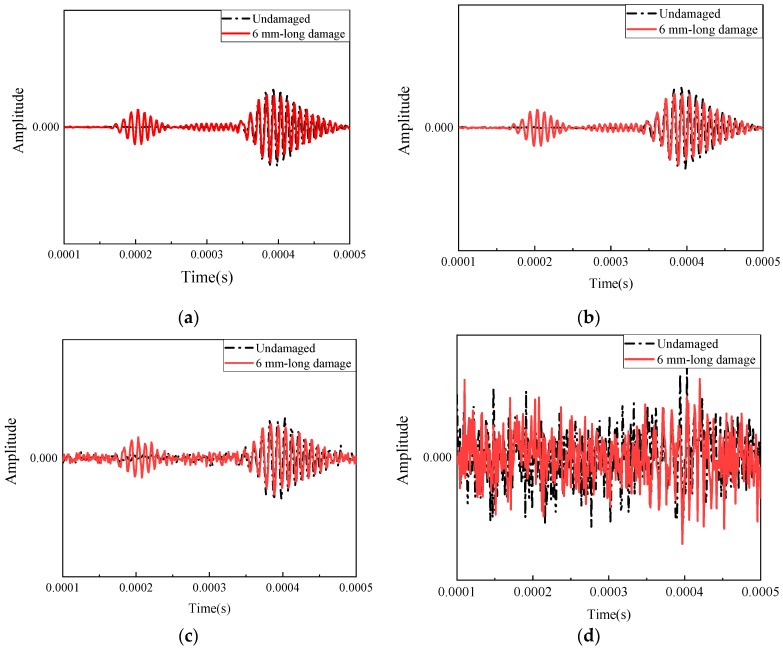
Noise level. (**a**) Original signal; (**b**) SNR = 120 dB; (**c**) SNR = 100 dB; (**d**) SNR = 80 dB.

Response data from sensors could easily be contaminated by noise. Noise was added to the collected signals based on the signal to noise ratio (SNR) that represents the ratio of the signal strength to the background noise strength as [31]:

(4) $$SNR_{dB}=10\log_{10}(\frac{P_{\mathrm{signal}}}{P_{\mathrm{noise}}})$$

where $P_{\mathrm{signal}}$ and $P_{\mathrm{noise}}$ are the average power of signal and noise by the dB scale, respectively. Five different noise levels, ranging from 80 to 120 dB, were selected to State # 1-17 for machine learning to check the sensitivity of the uncertainty due to noise, shown in Figure 8. Figure 8a represented the original signal of undamaged state and 6 mm-long damage state with damage reflected package and boundary reflected package. Figure 8b,c showed the signals with the SNR equal to 120 dB and 100 dB respectively. When SNR reduced to 80 dB, the damage package was difficult to identify.

## 4. Feature Representation and Classification using Machine Learning

### 4.1. Feature Extraction Methods

The Lamb wave exhibits apparently non-stationary and nonlinear behavior. Hence, selection of damage-sensitive features is crucial to assist the classification and prediction [32]. In addition, the robustness of the feature under noise is also an essential factor for selecting features [33]. In this study, features were extracted from frequency-, time- or time–frequency-domains, while those damage-sensitive features were then selected in accordance with feature selection methods. 

In the time domain, physics-based features play an important role in Lamb wave feature extraction. Amplitude, energy and the correlation coefficient are three features that can represent the wave characteristic. The amplitude was obtained by the peak value of the damage wave packet. The energy calculated by the root mean square of wave (RMS) in the damage part were defined as

(5) $$\mathrm{rms}\;=\sqrt{\frac{1}{n}\overset{n}{\underset{i=1}{\sum }}e_{i}^{2}}$$

where *n* is the number of data point and *e_i_* is the signal. The correlation coefficient under the damage state was used to compare with that of the health state.

In the frequency domain, the amplitude was extracted as the features. Fifty samples were generated randomly in each scenario by additive white Gaussian noise, which was used for feature extraction and feature selection. 

The time-frequency domain analyze is effective to track the change of a system and its nonlinear behavior and the conventional techniques are mostly encompassed by the wavelet transform. It shows a good deal of potential in nonstationary signals analysis due to excellent local zooming property of the wavelet. By shifting and dilating the mother wavelet, a particular set of function, the signal can be decomposed, which could preserve the temporal information. Meanwhile, the wavelet coefficients are obtained to weight the signal, which represents the feature of the signal.

**Figure 9:**
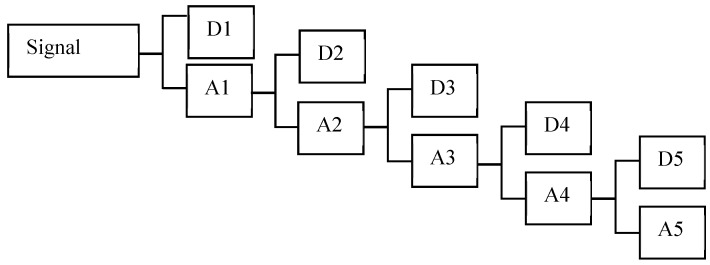
Full fifth level wavelet transform.

Discrete wavelet transforms analyze the signal through decomposing it into successive low and high frequency components. By implementing a wavelet filter of particular frequency band shifts along the time axis, DWT analyzes the signal, which makes the local examination of the signal become possible. The signal can be expressed as wavelet details and approximation in every level as shown [31]

(6) $$x\left(t\right)=\overset{n}{\underset{i=1}{\sum }}D_{i}\left(t\right)+A_{n}\left(t\right)$$

where $D_{i}\left(t\right)\;\mathrm{and}\;A_{n}\left(t\right)$ are the wavelet detail at the *i*thlevel and the wavelet approximation at the *n*thlevel. The frequency recursive relations are shown in Figure 9 for full 5thlevel wavelet decomposition, called the Mallat-tree decomposition. Meyer was chosen as the mother wavelet and six of the wavelet coefficients were applied as the damage sensitive features. 

### 4.2. Feature Selection and Criteria

The relief algorithm [34] is a supervised features selection method. Its simplicity and efficiency enable determining the sensitive features, especially for binary classification. Relief-F extended the relief into multiclass problems [35]. It randomly selects an observation from the training data and then searches for the most similar samples (near hit) in the same class and the nearest instances in different class (near miss). For each feature, it calculates the weights vector according to the absolute difference between the selected sample with the near hit and the near miss. To consider the incomplete or noisy data, which may cause the incorrect results, the relief-F selects *n* nearest hits and misses and calculates the average contributions to the weights instead of the single value. The differences between the values of attribute A for two observations *I*_1_ and *I*_2_, *diff*(*A*, *I_1_*, *I_2_*), were defined as [36]

(7) $$diff\left(A,I_{1},\;I_{2}\right)=\frac{\left|value\left(A,I_{1}\right)-value\left(A,I_{2}\right)\right|}{\max\left(A\right)-\min\left(A\right)}$$

and then, relief-F’s estimation *W*(*A*) was defined as the probabilities by the form:

(8) W(A)=P(different value of A|nearest instance from different class)−P(different value of A|nearest instance from same class)

As a result, the relief-F algorithm gave a weight to each feature depending on the feature’s ability to discriminate between samples in the different class. In this paper, this method was used to select the most sensitive feature among the different extracted features for further study.

### 4.3. Support Vector Machine for Classification

SVM is a powerful tool for classification in machine learning, which was developed by Vapnik [37]. The principle of SVM for classification is to construct a hyperplane that separates the data into two classes. It maps the input vector into a higher-dimensional feature space by applying kernel function (e.g., linear, polynomial or Gaussian radial basis function). An optimal hyperplane is then established in that feature space makes the separation by maximizing the margin from the hyperplane to the closest data points in either class.

Consider the set of training vectors $\left(x_{1},y_{1}\right)$, … $(x_{k},y_{k}),\in R^{N}$ belonging to two classes ($y_{i}=\left\{-1,\;1\right\}$). The aim is to look for the hyperplane to separate the data [38]:

(9) $$\left(w\cdot x\right)+b=0,\;w\in R^{N},\;b\in R$$

where w is the weight parameter controlling the orientation of the hyperplane and *b* is a scalar threshold adjusting the bias of margins between the optimal hyperplane and the support vectors [26]. Then the feature space for the linear classifier is shown

(10) f(x)=sgn((w·x)+b)

For the simplest case of a two-dimensional space, several linear classifiers could separate the data. The goal is to look for the hyperplane with largest margin, which is called the optimal hyperplane. Thus, all the training data are satisfying the constraints as follows [39]

(11) $$x_{i}\cdot w+b\geq +1\;\;\mathrm{for}\;y_{i}=+1$$

(12) $$x_{i}\cdot w+b\leq -1\;\;\mathrm{for}\;y_{i}=-1$$

The geometric distance from data point to hyperplane (**w**, *b*) is shown [40]

(13) $$d\left(\left(w,\;b\right),x_{i}\right)=\frac{y_{i}\left(x_{i}\cdot w+b\right)}{\left||w|\right|}\geq \frac{1}{\left||w|\right|}$$

To obtain the optimal hyperplane, the maximum distance to the closest data points should be found. From Equation (13), acquiring the maximum distance is the same as finding the minimum value of ||w||. Therefore, the optimization could also change into a convex quadratic programming problem [41]

(14) $$\mathrm{Minimize}\;\Phi \left(w\right)=\frac{1}{2}{\left||w|\right|}^{2}$$

The Lagrange multiplier is the main method to finding the local maxima and minima of a function subject to equality constraints. The problem is transformed into [41]

(15) $$L\left(w,b,\Lambda \right)=\frac{1}{2}{\left||w|\right|}^{2}-\overset{k\;}{\underset{i=1}{\sum }}\lambda_{i}\left[y_{i}\left(w^{T}x_{i}+b\right)-1\right]$$

where $\Lambda ={\left(\lambda_{1}\cdots \lambda_{k}\right)}^{T}$ are the Lagrange multiplier. The L(w,b,Λ) has to be minimized with respect to **w** and *b*, and maximized with respect to Λ≥0.

The decision function is given by [41]

(16) $$f\left(x\right)=\;\mathrm{sgn}\left(\overset{k\;}{\underset{i=1}{\sum }}\lambda_{i}^{*}y_{i}K\left(x,x_{i}\right)+b\right)$$

where the $K\left(x,x_{i}\right)$ is the kernel function, and three commonly used types are Gaussian radial basis function (RBF), polynomial function and sigmoid function [42]. In this paper, RBF was selected as the kernel function.

(17) $$K\left(x,x_{i}\right)=\exp\left(-\gamma {||x}_{i}-x_{i}||{}^{2}\right),\;\;\;\;\gamma >0$$

In general, the kernel function, shown in Equation (17), tends to construct a higher dimensional feature space and allows a projectile of data to this hyperplane to achieve being linearly separable. The kernel function helps SVM to be much more suitable for a different dataset, which can be used in non-linear classification. The different kernel functions have their applicability, including computation cost and parameter tuning. To enhance the accuracy of the damage prediction, it is important to select suitable penalty coefficient and kernel function parameter for the SVMs. 

Grid-search techniques (GS) were used to develop a set of optimal combination of the parameters (C, γ) in Equation (17). C is penalty coefficient adjusting the confidence interval range of the learning machine and γ is the kernel function parameter changing the mapping function [43]. The main steps of the GS method in SVM can be list. Firstly, gird search space needs to be built including the minimum and maximum value; then, through the orthogonal grid point matrix optimal value of parameter pairs (C, γ) are search and the fitness values of each point are calculated; finally, the best values of parameters C, γ can be use in SVM model for classification. Although the GS method would repeat the calculation for several times to search the optimal parameters, it is one of the simplest and most exhaustive methods and can parallelize the searching for these two independent parameters (C, γ) [33,44].

### 4.4. Assessment of Effectiveness of Learning Models using ROC Curves

The receiver operating characteristic (ROC) curves, which is generated by plotting the true positives rate against the false positives rate based on different thresholds, were employed as the evaluate tool in machine learning [33,45]. The area under the ROC curve (AUC) was summarized the degree or measure of separability. Consider that the method could be affected by skewed classes and insensitive to the change of class distribution, ROC was typically used in binary classification problems. Later, some researchers [46,47] extended it to multiclass classification by binarizing the labels. In this study, ROC curves were used to measure the ability of the SVM model, which identified the damages. 

## 5. Results and Discussion

### 5.1. Damage-Sensitive Features 

**Figure 10:**
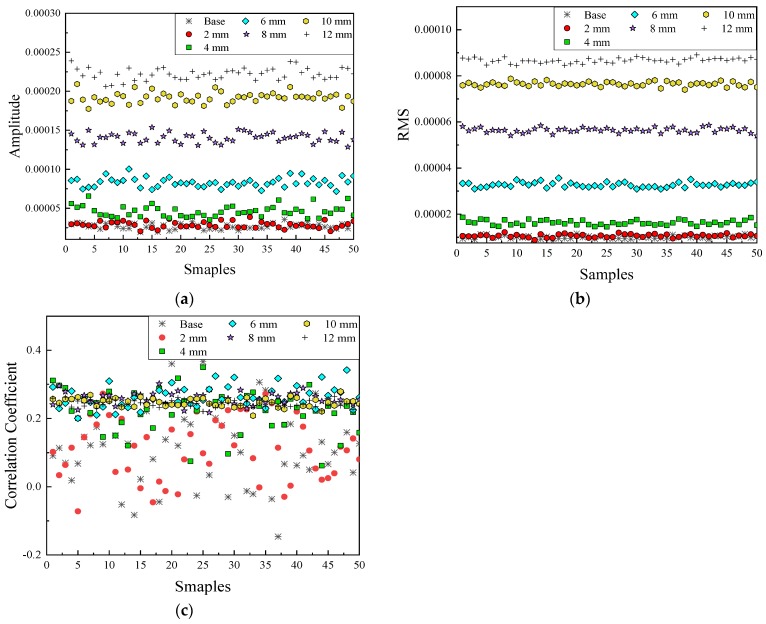
Features in the time domain. (**a**) Amplitude in time domain; (**b**) RMS in time domain; (**c**) Correlation coefficient in time domain.

**Figure 11:**
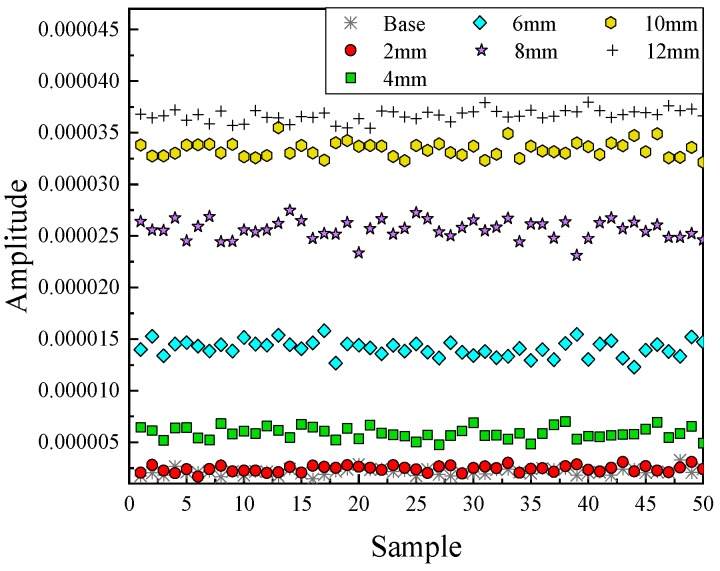
Features in the frequency domain.

**Figure 12:**
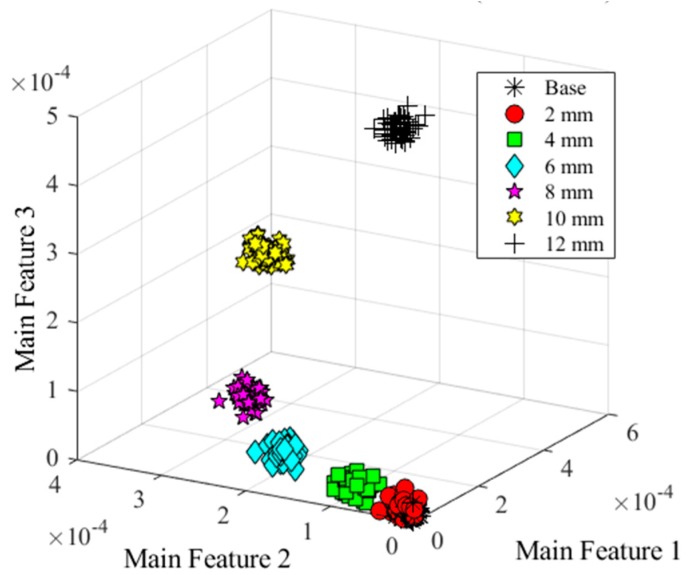
Features in the time–frequency domain.

This section was to address the effectiveness and sensitivity of the feature extraction and feature selection methods for capturing proper features for damage detection. Features from different domains were plotted in Figure 10, Figure 11 and Figure 12, in which six different damage states (State#2, #7 and #11 in Table 1) and the reference state (State #1) were displayed when the SNR was 100 dB. As illustrated in Figure 10a,c, three features were demonstrated in the time domain (i.e., amplitude, RMS and the correlation coefficient) and different symbols represented the different states for better visualizing different clusters.

Clearly, features using the amplitude, or the RMS provided distinguishable separation at most cases. Specifically, it seemed that RMS provided a better result than the amplitude, because several crossed data were occurred between 10 and 12 mm damages using the amplitude feature. Certain overlapping points were observed at the case of relatively small damage near the 2 mm-long damage state (in red circles in Figure 10a,b) with the reference in black asterisks. The results of the correlation coefficient were mixed together, which could not help to classify different states. As shown in Figure 11, the amplitude of the wave in frequency domain showed a similar result as the amplitude and RMS in the time domain, which was easy to distinguish the bigger damage (from 4 to 12 mm cases) and hard to separate the smaller cases with the references. 

The results of the wavelet coefficients were shown in Figure 12, where three of the wavelet coefficients were in a coordinate system. Clearly, most features were more distinguishable. Data in the state of base and 2-mm long damage were also much easier to separate. 

As stated, to quantitatively determine the quality of these features, Relief-F was used to rank the sensitive features in terms of their sensitiveness and the robustness, which is an individual evaluation filtering feature selection method for multilabel data [48]. It calculated the weights of features, which could estimate the quality and the relevance between each feature and target classification.

For each damage scenario, five different noise levels (which SNR were decreased from 120 to 80 dB) were considered to analyze the robustness of the features under noisy condition. In Table 2, 10 features were ranked by the relief-F method, including 6 wavelet coefficient features (W_1 to W_6), the amplitude of wave in time domain (Amp), the amplitude of the wave in the frequency domain (Frq), the correlation coefficient (Cor) and the RMS of the wave (RMS).

**Figure 13:**
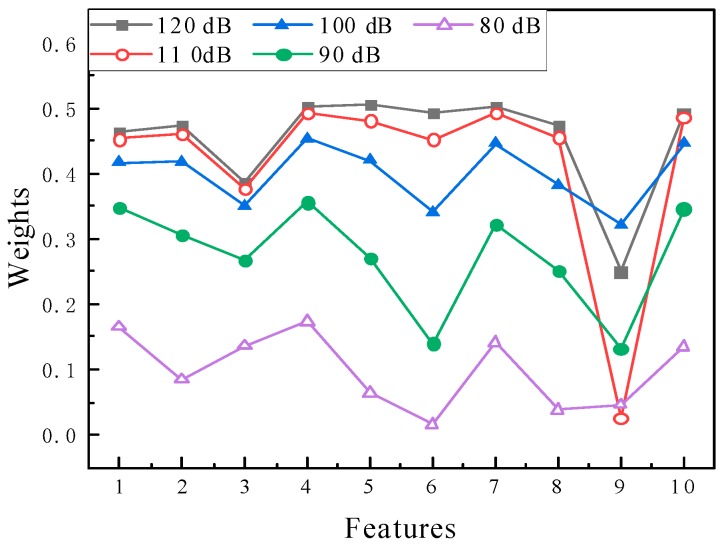
Weight of each feature under different noise levels.

The best features in different noise levels were W_5 and W_4 respectively, which were all belonging to wavelet coefficients. In contrast, the correlation coefficient (Cor) indicates the worst results, which was the same as in Figure 10. Figure 13 shows the weights of each feature. At a low noise level (SNR = 120 dB), W_5, W_4 and Amp had good performance for the classification, which shows the higher weights comparing with other features. When SNR decreased, all the feature weights declined, however, W_4 still maintained the highest weight. The wavelet coefficient could reduce the effects of the noise, which shows the stable high quality among other features. Hence these should be the most suitable features for further study. 

Although the physics-based features can classify the damage into different state at a low noisy environment (SNR = 100 dB), it is hard to get a high accuracy result under a higher noise level. Table 3 shows the result of the classification with a different method and features. Three physics-based features were used for classification by the traditional way respectively. Then, SVM was involved by three feature groups, including physics-based features (Amp, Frq, Cor and RMS), all feature pools (4 of the physics-based features and 12 of the wavelet coefficients) and the selected features by feature selection methods. 

By the traditional method, Amp, Frq and RMS presented the good result at 120 dB and 110 dB. However, with the level of SNR increased, the accuracy of the separation dropped down sharply. Specifically, only 39.43% of the data could be classified correctly through RMS, which was the highest one comparing with the Amp (19.43%) and Frq (34.86%). On the other hand, the SVM method showed superiority by the high dimensional features, which was much more accurate especially at high noise level states. In this method, the results were distinct by different feature groups. Using physics based features to train the data, although the accuracy reached 100% (SNR = 120 dB), the ratio began to reduce into 98.86% at 110 dB and then decreased to 53.17% at 80 dB. To increase the dimension of the features, all the features were used for training data. The accuracy of each state was not increased dramatically, which was lower than the model trained by selected features. Clearly, using selected features, 95.43% of the data was identified at 100 dB comparing with the 84% by all the features. In the case of SNR equal to 80 dB, nearly 17% of the accuracy was increased by feature selection. Therefore, SVM combining with the feature selection method can increase the accuracy of the classification.

### 5.2. Effectiveness and Sensitivity of the Feature Extraction Methods to Data Classification 

**Figure 14:**
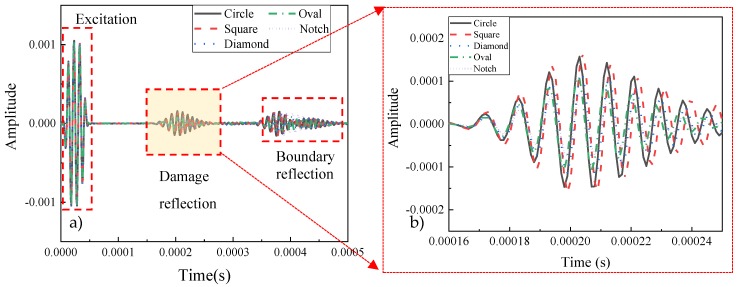
Received signals through the pitch-catch method.

The waves traveled through the plate and echoed when it arrived at the damage and boundary under different scenarios (shown in Figure 14a). In order to reduce the complexity of the signal, only the first three wave packets received by the receiver were used to analyze the characteristics of the signal. In the received signal, the first signal packet presented the excitation, which was the same in each scenario. The second packet collected at around 0.0002 s represented the echoed wave from the damage part, which certificated that the damage was located at the middle of the plate. Moreover, the third packet showed the echoed signal from the boundary away from the receiver. It is obvious that the wave gradually dispersed when it propagated further. Therefore, the second packet carried the information of the damage, which was chosen to extract the feature. 

The signals with different damage types were received respectively in Figure 14a. To analyze the received signal clearly, the signals were cut off at the second echoed signal, shown in Figure 14b. The amplitude of each signal was close. Moreover, the frequency of each signal was slightly different. The red short line represented the square-shaped damage had the highest amplitude (1.658 × 10^−4^) and lowest frequency. On the contrary, the green dotted line (oval-shaped damage) had the highest frequency. The notch-shaped damage had the lowest amplitude, which was 7.44 × 10^−5^.

**Figure 15:**
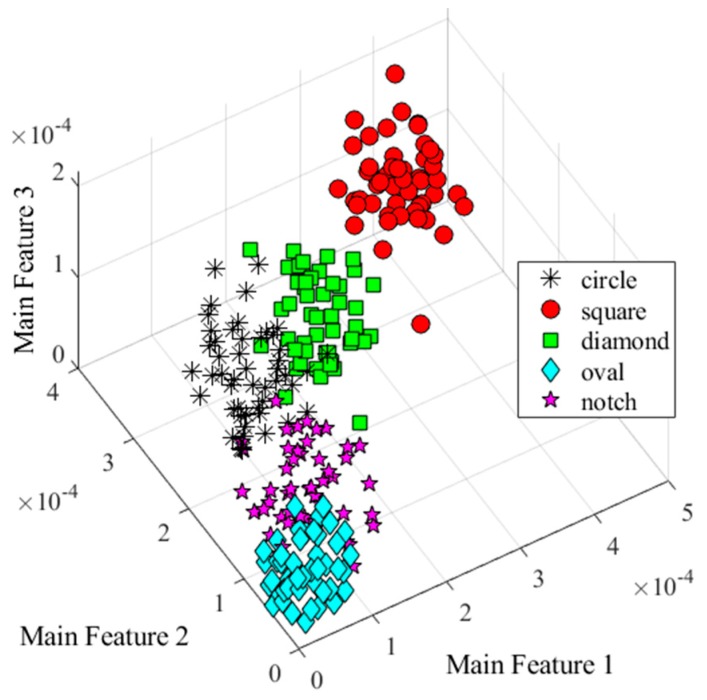
Damage features distribution (SNR = 90 dB).

From the result, six wavelet features were used to distinguish the damage types. To consider the interference of the noise and obtain more data, additive white Gaussian noise were added into the signal, which SNRs were set from 120 to 80 dB. Representing these data into the feature space, three of the wavelet features were selected to set a coordinate system. Figure 15 shows the feature clusters. Five different shapes represented the different damage types. Clearly, the features could separate the data into different damage shapes under SNR = 90 dB.

**Figure 16:**
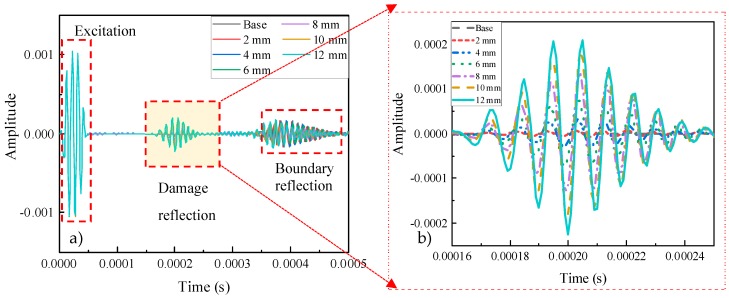
Received signals through the pitch-catch method.

The differences among these damage sizes were responded to in Figure 16b, which showed the detail of the damage part under different states. The frequencies of this part were similar when the length of damage changed. However, with the crack length increased, the amplitude of the received signal at the second packet was increased. Comparing the amplitude of the base state in the black short line and the 2-mm long damage in the red dash line, the difference between them was small, where the figures were 1.405 × 10^−6^ and 7.421 × 10^−6^ respectively. As the length of the damage increased from 4 to 10 mm, the magnitude of the difference in amplitude rose obviously. When damage equaled 12-mm long, the cyan-blue solid line had the highest amplitude, namely 2.246 × 10^−4^. 

**Figure 17:**
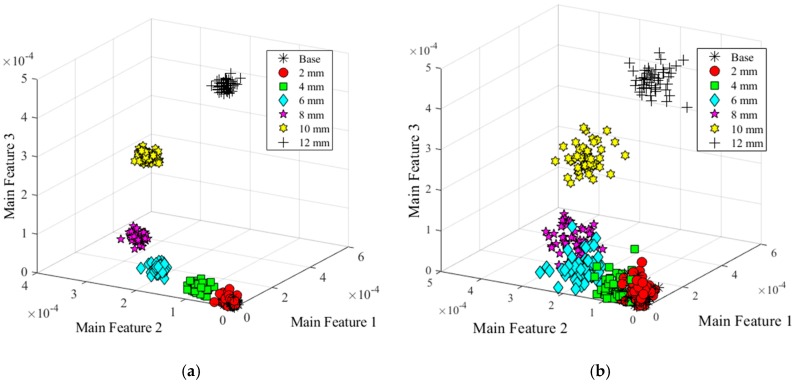
Feature distribution under two different noise levels. (**a**) Damage features distribution (SNR = 100 dB); (**b**) Damage features distribution (SNR = 90 dB).

As similar as the analysis of damage types, six wavelet coefficients were selected as the features under different noise levels. As clearly illustrated in Figure 17a,b, different colors and symbols manifested the data belonging to different states, which demonstrated the relationship between features and the classification of damage severities when the SNRs were equal to 100 dB and 90 dB respectively. In Figure 17a, data were clustered into seven groups. The black asterisk and red circular symbols indicated the data in the base state and 2-mm long damage state. From the results, these two sets were partially crossed. With the damage length increasing, the distances between these groups became more farther, especially for the damage with 10-mm and 12-mm long, which was easy to separate them into different states. However, the values of these features were divergent with the SNR decreasing, suggesting that the accuracy would be declining (shown in Figure 15b). The average deviation of these features was around 4 × 10^−10^, which was much bigger than that of 100 dB, about 4 × 10^−11^. Thus, it is hard to find thresholds to distinguish the data under the base state, 2-mm long damage state and 4-mm long damage state due to a part of them being mixed together. On the contrary, there was less effect for the larger damage state because of the greater distance between each one. Therefore, the features were extracted effectively and sensitively for classifying data.

### 5.3. Effectiveness of the Damage Type and Size to the Robustness of the Feature Captured

**Figure 18:**
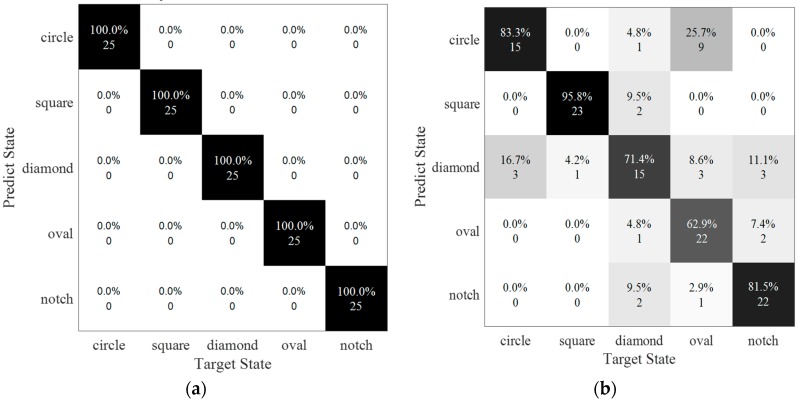
Classification of damage types under two noise levels. (**a**) SNR = 90 dB, accuracy = 100%; (**b**) SNR = 80 dB, accuracy = 77.6%.

SVM was used to classify the damage in 17 scenarios in terms of damage type, damage size and damage orientation. In each scenario, 50 sample data were randomly generated by the additive white Gaussian noise with the specific level where consists of five different levels as shown in Table 2. Under each noise level, 350 samples were involved in SVM model totally, which includes 50% of the data for training and the rest for testing. In Figure 18, confusion matrices, which represented the accuracy of the prediction by SVM, was shown when SNRs were 90 dB and 80 dB respectively. In Figure 18a, the classification was 100% correct by SVM. With the noise increase, the accuracy of the prediction was drop down sharply, which was only 77.6% in 80 dB. Of the circular-shaped damage 16.7% was misjudged into diamond. Diamond-shaped damage and oval-shaped damage had lower accuracy as 71.4% and 62.9%, respectively, which means that they were much easier to misjudge to other shapes.

**Figure 19:**
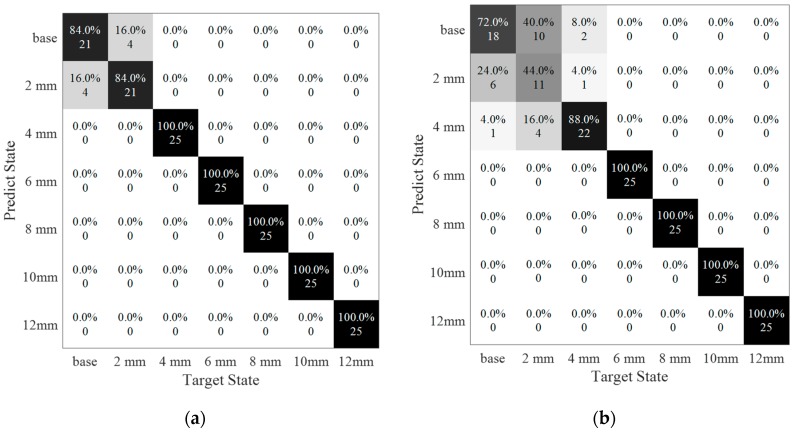
Classification of damage sizes under two noise levels. (**a**) SNR = 100 dB, accuracy = 95.4%; (**b**) SNR = 90 dB, accuracy = 86.3%.

In Figure 19, confusion matrices were used for representing the result of the classification by SVM as SNRs were set to 100 dB and 90 dB respectively. In these two matrices, the horizontal scale showed the target state of each data, and the vertical scale displayed the predicted result of the data-driven method.

In Figure 19a, the average accuracy for classifying the damage severity was equal to 95.43%, which means that most data were discriminated into the correct classifications. However, four of the samples belonging to 2-mm long damage were distributed into the base state. In addition, four data points in 2-mm long damage were detected into the base state. Increasing the noise level (SNR = 90 dB), the average accuracy was decreased to 86.29% (see Figure 19b). The errors were happened at small damage, especially on the base state and 2-mm long damage state. The target labels of the 25 samples were in the base state, of which six samples were misled into a 2-mm long damage state and one sample was misled into a 4-mm long damage state. In the damage condition of 2-mm long, 44% of the data was predicted with a true label, 40% sample was misinformed into base state and 16% of them was in a 4-mm long damage state with a false label. Similarly, two samples with 4-mm long damage had been misjudged as a base state and one misled into a 2-mm damage state. In addition, the rest of the data were detected into the correct labels.

**Figure 20:**
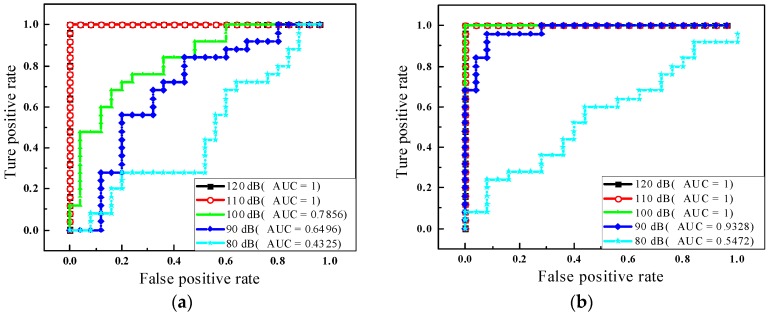
The ROC curves for multiclass. (**a**) Comparsion between State #1 and #7; (**b**) Comparsion between State #1 and #8.

Despite that the average accuracy of classification under the specific noise level was high, the confusion between the small damage and reference state occurred frequently. Hence, the ROC curves in Figure 20 provided the reference for the accuracy of categorizing the base state and small damage states (2mm-long damage and 4mm-long damage state) under different noise levels. Figure 20a depicted the ROC curves only considering the base state and 2mm-long damage state when SNRs were from 80 to 120 dB respectively. As usual, the more the ROC curve is tilted towards the left, the larger the AUC values are, which means that the result is much more acceptable. In the 120 dB level and 110 dB level, the classifications were entirely correct that the AUCs were equal to 1. It dropped to 0.7856 while the noise level changed to 100 dB. The previous study [49] confirmed that the prediction is unacceptable if the AUC is lower than 0.75. Obviously, when the noise level increased to 90 dB and 80 dB, the values of AUCs were 0.6496 and 0.4325, respectively, which fell in unacceptable zones. As shown in Figure 20b, the results of comparing the base state with a 4mm-long damage state were quite better than that the previous one. Specifically, the precise predictions were obtained when SNRs were from 120 to 100 dB. The AUC was equal to 0.9328 due to the noise level of SNR = 90 dB. Additionally, it is lower than the threshold of 0.75 at the 80 dB situation.

### 5.4. Effectiveness of Damage Orientation to the Robustness of the Feature Captured

**Figure 21:**
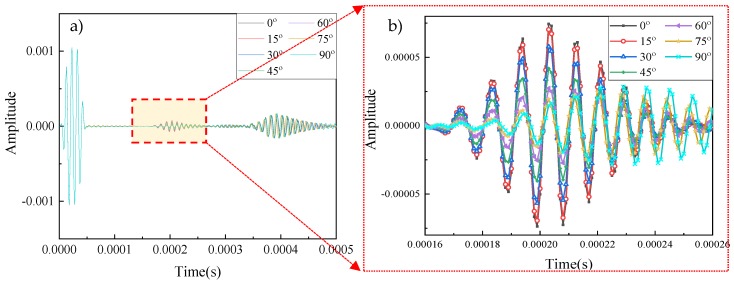
Received signals through the pitch-catch method (**a**) and (**b**).

Detecting the orientation of damage is also a significant and difficult issue in Lamb wave-based health monitoring. The 6-mm long notch-shaped damage was rotated 15-degree clockwise each time so that seven different orientations were set up. The received signals from the finite element model were plotted in Figure 21. The second package brought the information about the damage, and the frequencies of the waves in this part were similar. Figure 21b showed the details of the signals, which illustrated that the amplitudes of the signals were reduced from 7.439E-5 to 1.658E-5 as the angle of damage increasing from 0 to 90° because of the vertical projected area decreased. As the horizontal projection increased, the length of the wave packet was climbing. The black solid line represented the initial state (6mm-long damage without rotation), which had the highest peak value and the packet was from 0.00016 to 0.00026 s. As for the cyan-blue dotted line presenting the 6mm-long damage with 90-degree orientation, the amplitude was the smallest, but the packet length increased. 

**Figure 22:**
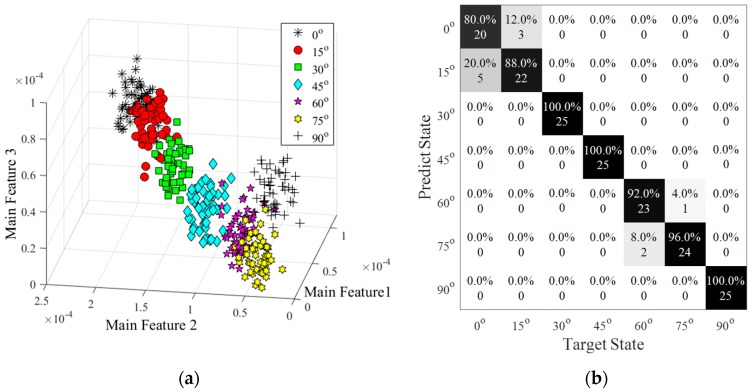
Damage identification results. (**a**) Feature distribution; (**b**) Confusion matrix.(accuracy = 93.7%, SNR = 100 dB).

Figure 22a showed the result of the feature distribution under 100 dB. Wavelet coefficient features clearly classified these data into seven different groups, which distributions were quite different from the damage severity. The distances between each group were similar. The result of the identification was shown in Figure 22b. Different from the damage severity, the misleading might happen in any of the state. For instant, five of the base state samples were misled into the 15-degree rotation state, and three of the 15-degree rotation state data were misjudged as the base state. In the rest of the states, most of the predictions were allocated into the target state, except two misleading in a 60-degree rotation and one misled in a 75-degree rotation state. The accuracy of the SVM classification was 93.71%, which was a little lower than the classification in damage severity.

## 6. Further Discussion of Structural Uncertainty Related to Engineering Applications

Clearly, engineering structures are often exposed to a complex environment with high levels of uncertainty. As a result, Lamb wave signals collected from complex structural systems in fields could be highly affected by structural uncertainty, which in turn affects the effectiveness of the proposed methods for engineering applications. As a part of this study, further discussion was presented herein to address the effectiveness of the proposed methods when handling structural uncertainty due to mixed data types, noise level and material discontinuity from weldment that engineers often face with in field. Note that though there are a wide range of high variances of uncertainty, we narrowed our work to three common issues for simplicity to demonstrate the methods.

### 6.1. Impacts of Structural Uncertainty due to Noise Interferences to the Robustness of Data Classification

One of the greatest challenges in signal processing is uncertainty from noise (e.g., measuring noise from sensor systems and environmental noise) that usually interferes with raw data. State #1-17 were designed under five different noise levels, which the SNRs were equal from 80 to 120 dB as listed in Table 2, to address how effectively and sensitively the features gained by feature extraction methods respond under a certain noise level. 

**Figure 23:**
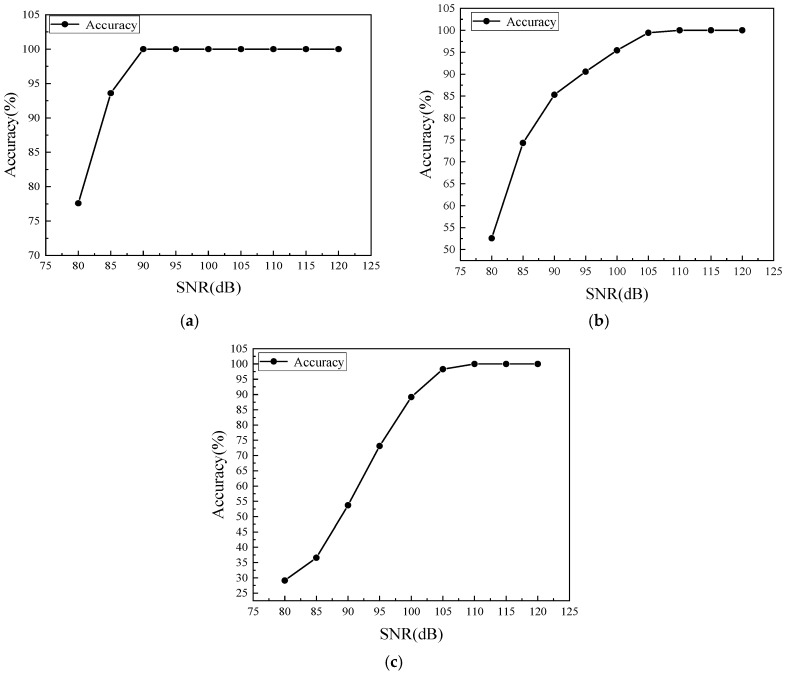
Damage identification to noise interference. (**a**) Damage type; (**b**) Damage size; (**c**) Damage orientation.

Figure 23a,c showed the accuracies of the damage identification in different noise levels under four conditions. They all represented that when the SNR was higher, the accuracies of the classification were increased. In the damage type prediction, the accuracy reached 100% when the SRN was higher than 90 dB. However, the accuracy was dropped down dramatically at 80 dB. At the same level, only 52.57% of the data was distinguished into the target state when classified into a different damage size. When the SNR was bigger than 105 dB, the accuracy reached 100%. At the orientation identification analysis, the value was lower than the damage size analysis, which just had 29.14% of the data classified into the target label at 80 dB. When the SNR increased, the accuracy was rising dramatically, which was 53% at 90 dB and 89.14% at 100 dB. The SNR arrived 100% at 110 dB. Therefore, the noise was a critical issue to affect the classification results directly. When SNR was 110 dB or above, the prediction was precise in all conditions. However, when SNR approached 80–90 dB, the wave packet that carried the information of the damage would be overlapped by noise, so that it was hard to distinguish their target state. 

### 6.2. Impacts of Structural Uncertainty due to Mixed Data Types to the Robustness of Data Classification 

Collected data could be mixed in more complex conditions with different damage orientation, damage level and damage types. Therefore, this section was to use trained models to classify the mixed data types with 325 data points in a total of 13 states, including different levels of damage size and orientation together, as shown in Table 2.

**Figure 24:**
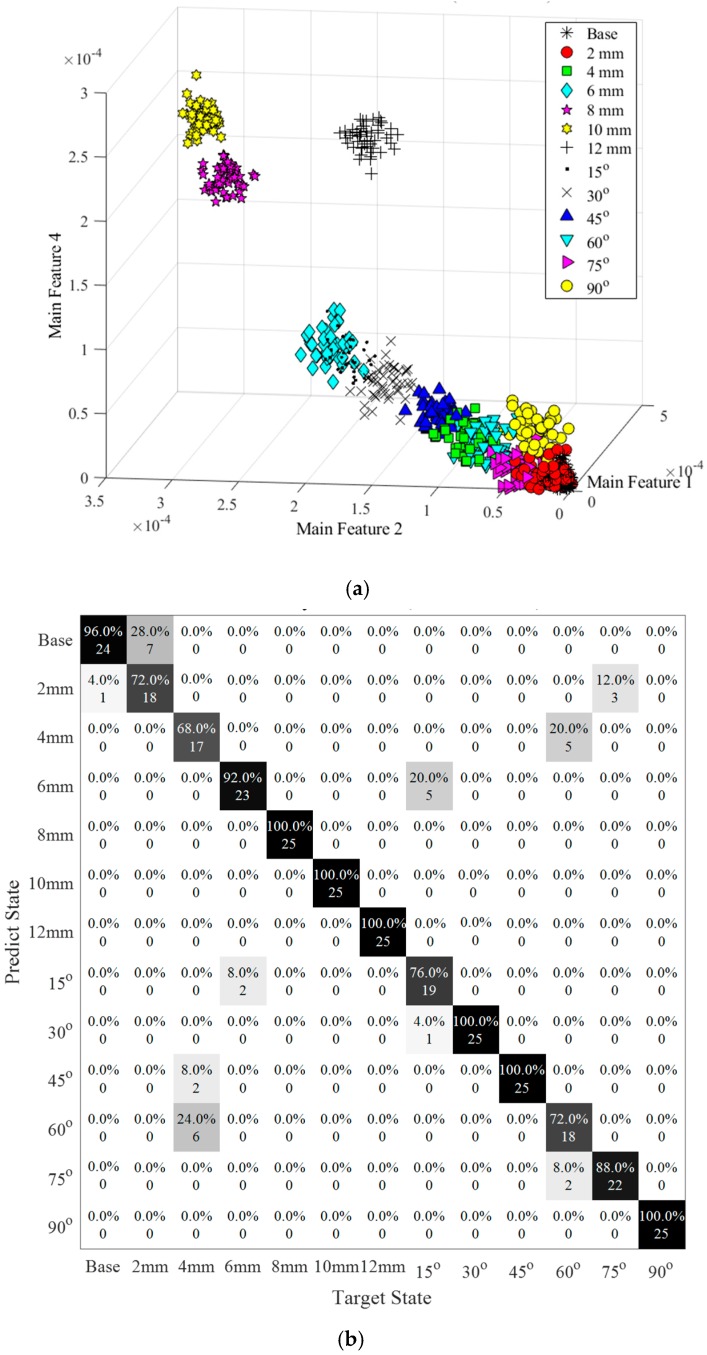

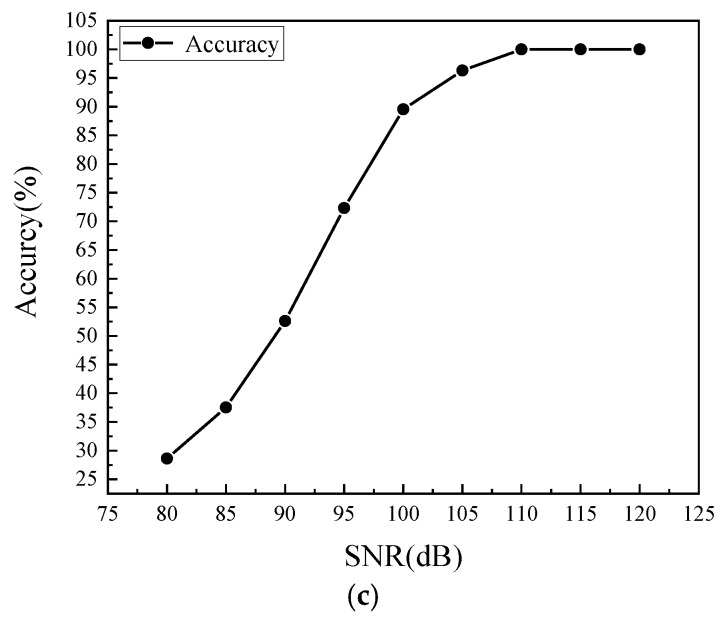
Data classification for mixed data types: (**a**) Feature distribution (SNR = 100 dB); (**b**) Confusion matrix (accuracy = 89.54% SNR = 100 dB); (**c**) Noise interference.

Figure 24a demonstrated the feature extraction in different states when SNR was equal to 100 dB that most of the data could be grouped by the wavelet coefficients. Specifically, the cluster of different damage orientations were situated between the 6mm-long damage group and 2mm-long damage group, which proved that the vertical projected area of the damage was the essential factor to determine the features’ value. The feature of damages with a 15-degree, 30-degree and 45-degree feature were placed between 6mm-long and 4mm-long damage, and the 60-degree, 75-degree and 90-degree feature were located between 4mm-long and 2mm-long damage. Some overlaps appeared between adjacent clusters causing the accuracy of the classification reduced.

Figure 24b illustrated the SVM classification results among 13 different states under SNR equaling to 100 dB. The average accuracy was 89.54%. The misleading between the base state and State #7 still existed, which had 4% of the data belonging to the base state predicted into State #7 and 28% of the data should be State #7 predicted into the base state. Moreover, 8 of the 25 samples in the 4 mm-long damage state were misled to the wrong state, including 2 for the 45-degree state and 4 for the 60-degree state respectively. Similarly, the 60-degree state was much easier to confuse with the 4mm-long damage state containing five wrong predictions. In addition, 6 mm-long damages were similar to the damage rotated 15-degree leading to 20% of samples being misjudged and in the 15-degree damages data, 8% of the samples being misjudged. Figure 24c exhibited the accuracy of classification when having a mixed damage size and orientation identification. Clearly, a similar trend was observed as stated in Figure 23a,c. When SNR was equal to 80 dB, only 28.62% of the data could be detected into the correct label. Increasing the SNR, the accuracy increased to 52.62% at 90 dB and 89.54% at 100 dB. 

Although the accuracy of classification considering the damage size and orientation together was lower than that of the individual analysis of damage size or orientation, this method was enough to guide us to understand damages in detail. 

### 6.3. Impacts of Structural Uncertainty due to Material Discontinuity from Weldment to the Robustness of Data Classification

**Figure 25:**
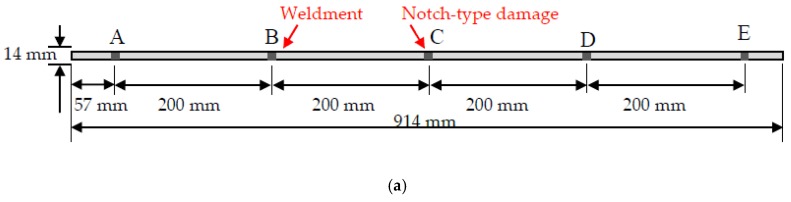

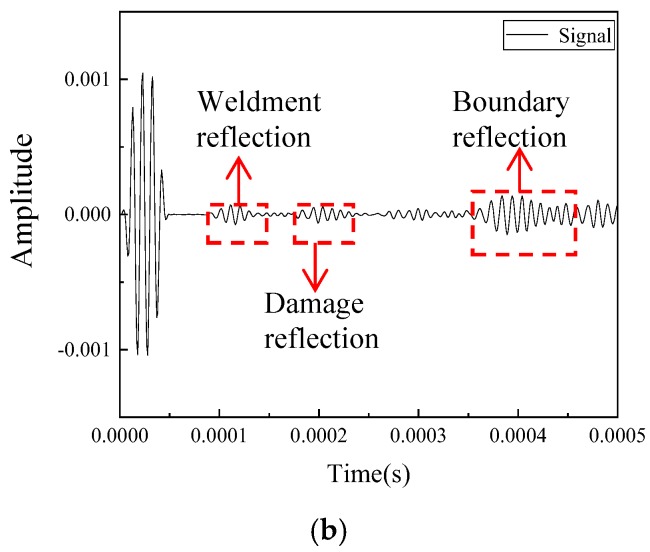
Plate with weldment and 6 mm-long notch-type damage. (**a**) Plate with a butt welded joint at point B and a 6-mm long notch-type damage at point; (**b**) Signal collected from point A.

Material discontinuity due to weldment creates more complexity for the lamb wave signal process. This section was to discuss the effectiveness of the proposed method for classifying such structural uncertainty. To test the accuracy of this model, a new dataset was built by the numerical simulation method. An identical plate, illustrated in Figure 4, was modified by adding a butt weldment at the location of point B and a 6mm-long notch at the location of point C, shown in Figure 25a. The width of the weldment was 5 mm and the welding filler was Ti-6Al-4V. To enlarge the data, 175 signals were augmented using white Gaussian noise with different levels. 

**Figure 26:**
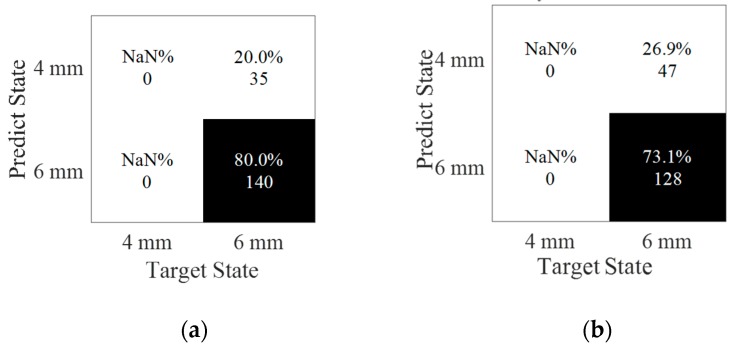
Classification of damage sizes under two noise levels. (**a**) SNR = 100 dB, accuracy = 80.0%; (**b**) SNR = 90 dB, accuracy = 73.1%.

With the interaction of the weldment, the received signal had more reflected packages than that of the previous one, shown in Figure 25b. From the signal, the reflections came from the weldment, damage and the boundary. The label of each data was predicted by SVM, which was trained in Section 5. Table 4 showed the comparation of the predicted result in a 6 mm-long damage and the one added in the weldment. Clearly, the prediction of the damage with the weldment posed a challenge in classification as compared to cases without weldment. Specifically, in most of the cases (shown in Figure 26), 6-mm long damage was classified accurately, which expected 100% of the SNR to equal 80 dB. With the weldment present, it interfered with the signal and reduced the accuracy of the prediction. However, most of the damage could be tested by this model. A total of 80% of damaged cases were classified into a 6 mm-long damage group, and 20% of the damage was predicted as 4 mm-long at 100 dB. The misleading increased to 26.9% when the noise level approached 90 dB.

## 7. Conclusions

This study investigated the Lamb wave-based damage detection method enriched by machine learning to accelerate classification associated with damage size and orientation. The dataset of the Lamb wave propagation through the aluminum beam was generated using a simulation. Different features under different domains were extracted and evaluated using feature selection methods. Impacts of noise interference to the effectiveness of the methods were also addressed. Some conclusions can be drawn as follows:

(a) The learning framework provided a workflow from dataset generation, to sensitive feature extraction and to prediction model for lamb-wave-based damage detection. Note that although SVM learning algorithms were designed for learning model training, the deep learning could be deployed in future work.
(b) Different features generated from different domains could provide various levels of sensitivity to damage. With the aid of feature selection, time-frequency features and wavelet coefficients, the highest damage-sensitivity was exhibited, as compared to the features in either the time domain or frequency domain. These features that contained the information in both time- and frequency-domains were also much more robust to noise.
(c) The results in the case study demonstrated that the SVM method could effectively classify the damage type and damage size to certain noise levels, near 110 dB. With an increase of noise to 80 dB, the accuracy of the classification dramatically dropped to 57%.
(d) The damage orientation could be classified using features of wavelet coefficients. The accuracy of the result was slightly lower than that of the damage size, mainly because the signals generated by the finite element method were from a one-dimensional propagation of the wave, and thus lacked the entire spatial information about the two-dimensional orientation. For further studies, two-dimensional wave propagation could be used for more accurate spatial information about the damage.
(e) Noise interference, mixed data types, the level and material discontinuity from weldment were used to address the structural uncertainty and their impacts to the effectiveness of the proposed methods. The findings revealed that the proposed methods were still effective for data classification. Further research will be carried out, as noise is still a big challenge when its level reached up to a similar order as to where the signals were, while material discontinuity from weldment also posed a complex situation for classification.

## Figures and Tables

**Table 1:** Test matrix for computation modeling.

Case	Label	Damage Type	Damage Size	Damage Orientation	Noise Interference
Reference	State #1	/	/	/	Noise levels of from 80 dB to 120 dB
Variance due to damage type	State #2	Notch-shaped damage	6-mm long	90 degree	
	State #3	Circular-shaped damage	6-mm diameter	/	
	State #4	Square-shaped damage	6-mm long	/	
	State #5	Diamond-shaped damage	6-mm long	/	
	State #6	Oval-shaped damage	6-mm long	/	
Variance due to damage size	State #7	Notch-shaped damage	2-mm long	90 degree	
	State #8	Notch-shaped damage	4-mm long	90 degree	
	State #2	Notch-shaped damage	6-mm long	90 degree	
	State #9	Notch-shaped damage	8-mm long	90 degree	
	State #10	Notch-shaped damage	10-mm long	90 degree	
	State #11	Notch-shaped damage	12-mm long	90 degree	
Variance due to damage orientation	State #12	Notch-shaped damage	6-mm long	0 degree	
	State #13	Notch-shaped damage	6-mm long	15 degree	
	State #14	Notch-shaped damage	6-mm long	30 degree	
	State #15	Notch-shaped damage	6-mm long	45 degree	
	State #16	Notch-shaped damage	6-mm long	60 degree	
	State #17	Notch-shaped damage	6-mm long	75 degree	
	State #2	Notch-shaped damage	6-mm long	90 degree	

**Table 2:** Feature Selection.

Noise Level	Feature Rank									
	1st	2nd	3rd	4th	5th	6th	7th	8th	9th	10th
120 dB	*W_5*	*W_4*	*Frq*	*W_6*	*RMS*	*W_2*	*Amp*	*W_1*	*W_3*	*Cor*
110 dB	*W_4*	*Frq*	*RMS*	*W_5*	*W_2*	*Amp*	*W_1*	*W_6*	*W_3*	*Cor*
100 dB	*W_4*	*RMS*	*Frq*	*W_5*	*W_2*	*W_1*	*Amp*	*W_3*	*W_6*	*Cor*
90 dB	*W_4*	*W_1*	*RMS*	*Frq*	*W_2*	*W_5*	*W_3*	*Amp*	*W_6*	*Cor*
80 dB	*W_4*	*W_1*	*Frq*	*W_3*	*RMS*	*W_2*	*W_5*	*Cor*	*Amp*	*W_6*

**Table 3:** Accuracy of different features.

Method		Classification by Physics-Based			Classification by SVM		
**Features**		*Amp*	*Frq*	*RMS*	No feature selection		Feature selection
					Physics based Features	All Features	Selected features (wavelet coefficients)
**Noise level**	120 dB	100.00%	100.00%	100.00%	100.00%	100.00%	**100.00%**
	110 dB	97.71%	100.00%	98.86%	98.86%	98.86%	**100.00%**
	100 dB	81.14%	86.29%	84.00%	92.00%	84.00%	**95.43%**
	90 dB	44.00%	64.00%	72.00%	80.00%	72.00%	**86.29%**
	80 dB	19.43%	34.86%	39.43%	53.71%	39.43%	**56.00%**

**Table 4:** Prediction.

Noise Level	120 dB	110 dB	100 dB	90 dB	80 dB
Without weldment	100.00%	100.00%	100.00%	100.00%	56.4%
With weldment	100.00%	100.00%	80.00%	73.10%	65.71%

## References

[1] Giurgiutiu V. (2007). Structural Health Monitoring: With Piezoelectric Wafer Active Sensors.

[2] Feng D., Feng M.Q., Monitoring H. (2016). Vision-based multipoint displacement measurement for structural health monitoring. Struct. Control. Health Monit..

[3] Pan H., Zhang Z., Wang X., Lin Z. Image-Based Damage Conditional Assessment of Large-Scale Infrastructure Systems using Remote Sensing and Deep Learning Approaches. Proceedings of the 2019 TechConnect World Innovation Conference.

[4] Doebling S.W., Farrar C.R., Prime M.B. (1998). A summary review of vibration-based damage identification methods. Shock Vib. Dig..

[5] Kong X., Cai C.-S., Hu J. (2017). The state-of-the-art on framework of vibration-based structural damage identification for decision making. Appl. Sci..

[6] Kong X., Cai C., Kong B. (2014). Damage detection based on transmissibility of a vehicle and bridge coupled system. J. Eng. Mech..

[7] Mitra M., Gopalakrishnan S. (2016). Guided wave based structural health monitoring: A review. Smart Mater. Struct..

[8] Ahmed M.N. (2014). A Study of Guided Ultrasonic Wave Propagation Characteristics in Thin Aluminum Plate for Damage Detection.

[9] Saravanos D.A., Heyliger P.R. (1995). Coupled layerwise analysis of composite beams with embedded piezoelectric sensors and actuators. J. Intell. Mater. Syst. Struct..

[10] Seale M.D., Smith B.T., Prosser W.H. (1998). Lamb wave assessment of fatigue and thermal damage in composites. J. Acoust. Soc. Am..

[11] Hu N., Shimomukai T., Fukunaga H., Su Z. (2008). Damage identification of metallic structures using A0 mode of Lamb waves. Struct. Health Monit..

[12] Xu B., Giurgiutiu V. (2007). Single mode tuning effects on Lamb wave time reversal with piezoelectric wafer active sensors for structural health monitoring. J. Nondestruct. Eval..

[13] Kong X., Cai C., Kong B. (2016). Numerically extracting bridge modal properties from dynamic responses of moving vehicles. J. Eng. Mech..

[14] Kong X., Cai C., Deng L., Zhang W. (2017). Using dynamic responses of moving vehicles to extract bridge modal properties of a field bridge. J. Bridge Eng..

[15] Nair A., Cai C., Kong X. (2019). Acoustic emission pattern recognition in CFRP retrofitted RC beams for failure mode identification. Compos. Part B Eng..

[16] Pan H., Gui G., Lin Z., Yan C. (2018). Deep BBN learning for health assessment toward decision-making on structures under uncertainties. KSCE J. Civ. Eng..

[17] Zhang Z., Wang X., Pan H., Lin Z. Corrosion-Induced Damage Identification in Metallic Structures using Machine Learning Approaches. Proceedings of the 2019 Defense TechConnect Innovation Summit.

[18] Zhang Z., Pan H., Lin Z. Data-Driven Identification for Early-Age Corrosion-Induced Damage in Metallic Structures. Proceedings of the Bridge Engineering Institute Conference 2019 (BEI-2019).

[19] Pan H., Ge Y., Lin Z. AI-Enabled Disaster Assessment and Resilience for Interdependent Critical Civil Infrastructures. Proceedings of the 2019 Defense TechConnect Innovation Summit.

[20] Wang W., Bao Y., Zhou W., Li H. (2018). Sparse representation for Lamb-wave-based damage detection using a dictionary algorithm. Ultrasonics.

[21] Yang J., He J., Guan X., Wang D., Chen H., Zhang W., Liu Y. (2016). A probabilistic crack size quantification method using in-situ Lamb wave test and Bayesian updating. Mech. Syst. Signal Process..

[22] Legendre S., Massicotte D., Goyette J., Bose T.K. (2001). Neural classification of Lamb wave ultrasonic weld testing signals using wavelet coefficients. IEEE Trans. Instrum. Meas..

[23] Su Z., Ye L. (2004). Lamb wave-based quantitative identification of delamination in CF/EP composite structures using artificial neural algorithm. Compos. Struct..

[24] Das S., Chattopadhyay A., Srivastava A.N. (2010). Classifying induced damage in composite plates using one-class support vector machines. AIAA J..

[25] Sun F., Wang N., He J., Guan X., Yang J.J. (2017). Lamb wave damage quantification using GA-based LS-SVM. Materials.

[26] HosseinAbadi H.Z., Amirfattahi R., Nazari B., Mirdamadi H.R., Atashipour S.A. (2014). GUW-based structural damage detection using WPT statistical features and multiclass SVM. Appl. Acoust..

[27] Su Z., Ye L. (2009). Identification of Damage Using Lamb Waves: From Fundamentals to Applications.

[28] Achenbach J. (2012). Wave Propagation in Elastic Solids.

[29] Yang M., Qiao P. (2005). Modeling and experimental detection of damage in various materials using the pulse-echo method and piezoelectric sensors/actuators. Smart Mater. Struct..

[30] Giurgiutiu V., Bao J., Zhao W. (2003). Piezoelectric wafer active sensor embedded ultrasonics in beams and plates. Experimental mechanics Exp. Mech..

[31] Lin Z., Pan H., Wang X., Li M. (2018). Data-driven structural diagnosis and conditional assessment: From shallow to deep learning. Sensors and Smart Structures Technologies for Civil, Mechanical, and Aerospace Systems 2018.

[32] Pan H., Lin Z., Gui G. (2019). Enabling damage identification of structures using time series–based feature extraction algorithms. J. Aerosp. Eng..

[33] Gui G., Pan H., Lin Z., Li Y., Yuan Z. (2017). Data-driven support vector machine with optimization techniques for structural health monitoring and damage detection. KSCE J. Civ. Eng..

[34] Kira K., Rendell L.A. (1992). The Feature Selection Problem: Traditional Methods and a New Algorithm.

[35] Kononenko I., Šimec E., Robnik-Šikonja M. (1997). Overcoming the myopia of inductive learning algorithms with RELIEFF. Appl. Intell..

[36] Stief A., Ottewill J.R., Baranowski J. (2018). Relief F-based feature ranking and feature selection for monitoring induction motors. 2018 23rd International Conference on Methods & Models in Automation & Robotics (MMAR).

[37] Vapnik V. (2013). The Nature of Statistical Learning Theory.

[38] Dibike Y.B., Velickov S., Solomatine D., Abbott M.B. (2001). Model induction with support vector machines: Introduction and applications. J. Comput. Civ. Eng..

[39] Burges C.J.C. (1998). A tutorial on support vector machines for pattern recognition. Data Min. Knowl. Discov..

[40] Boswell D.J., Diego E. Introduction to Support Vector Machines. http://www.work.caltech.edu/~boswell/IntroToSVM.pdf.

[41] Burbidge R., Buxton B.J. An Introduction to Support Vector Machines for Data Mining. http://www.cs.ucl.ac.uk/staff/r.burbidge/pubs/yor12-svm-intro.html.

[42] Santos A., Figueiredo E., Silva M., Sales C., Costa J.C.M.A. (2016). Machine learning algorithms for damage detection: Kernel-based approaches. J. Sound Vib..

[43] Han S., Qubo C., Meng H. (2012). Parameter selection in SVM with RBF kernel function. World Automation Congress 2012.

[44] Hsu C.-W., Chang C.-C., Lin C.-J. A Practical Guide to Support Vector Classification. https://www.researchgate.net/profile/Chenghai_Yang/publication/272039161_Evaluating_unsupervised_and_supervised_image_classification_methods_for_mapping_cotton_root_rot/links/55f2c57408ae0960a3897985/Evaluating-unsupervised-and-supervised-image-classification-methods-for-mapping-cotton-root-rot.pdf.

[45] Pan H., Azimi M., Yan F., Lin Z. (2018). Time-frequency-based data-driven structural diagnosis and damage detection for cable-stayed bridges. J. Bridge Eng..

[46] Wang Z. (2018). Deep learning-based intrusion detection with adversaries. IEEE Access.

[47] Shah M., Wang D., Rubadue C., Suster D., Beck A. (2017). Deep learning assessment of tumor proliferation in breast cancer histological images. 2017 IEEE International Conference on Bioinformatics and Biomedicine (BIBM).

[48] Urbanowicz R.J., Meeker M., La Cava W., Olson R.S., Moore J.H. (2018). Relief-based feature selection: Introduction and review. J. Biomed. Inform..

[49] Fan J., Upadhye S., Worster A. (2006). Understanding receiver operating characteristic (ROC) curves. Can. J. Emerg. Med..

